# Palestine/the Holy Land, 1922, a ‘laboratory’ for sustainable malaria control

**DOI:** 10.5281/zenodo.20677727

**Published:** 2026-06-13

**Authors:** Anton Alexander

**Affiliations:** 1 BC Business Centrum, Elscot House, Arcadia Avenue, London N3 2JU, United Kingdom.

## Abstract

Until recently, the main cause of concern for those involved with malaria elimination was the emergence of insecticide and drug resistance. However, since the withdrawal of USA funding for malaria control, as well as dwindling foreign aid budgets of European countries, the additional concern that now exists is of a lack of finance in support of that work. This paper sets out to remind that with only limited finance and without reliance on insecticides, drugs or bednets, the launch took place of successful sustainable malaria control as far back as 1922, and the principles which were then applied are available for use elsewhere.

On 22^nd^ April 2026, during a webinar hosted jointly by APMEN and NAVBD entitled ‘Malaria: Global Trends, Regional Insights and Country Action Plans’, a participating panellist, Dr. Andrea Bosman of the World Health Organization (WHO), reminded us of the sobering situation we are facing. He described a rapid, global deterioration of the malaria situation, which included the threats of a financial cataclysm of a shrinking/shifting of major funding, and also the threats of biological widespread insecticide and emerging drug resistance [1].

Previously, the emphasis for a pessimistic malaria outlook had been primarily directed at the failure of tools, such as insecticide and drug resistance or faltering of rapid diagnostics [2,3]. Now, with the withdrawal (USA) or reduced funding (EU), the picture has changed to two significant threats – reduced finance and failing tools – and has brought to mind something overlooked.

The 2012 WHO Handbook on Integrated Vector Management [4] included a brief historical note about malaria elimination. It stated: "*Before the Second World War, … elimination of* [malaria] *was never on the agenda...*“. This is not strictly correct. Events in Palestine appear to have been overlooked by WHO when compiling its historical note because in 1922, Palestine was to become the place for the first launch anywhere of successful sustainable malaria control, the phase immediately preceding malaria elimination.

Lack of finance was a huge factor in shaping the 1922 Palestine malaria control method. Further, the method was not reliant on insecticides. The above extract from the WHO Handbook continues “*The advent of DDT and other organochlorine pesticides during the 1940s changed this situation, …*”, confirming these insecticides and drugs did not exist in Palestine in 1922.

There exists, therefore, a proven method for successful and sustainable malaria control that was launched in Palestine in 1922, with only limited or reduced finance and was not reliant either on drugs, insecticides or bednets.

Clearly, the 1922 Palestine approach deserves a closer examination.

## Background to the 1922 malaria control

Conditions in Palestine in 1922 were, in fact, ideal for a controlled experiment that attempted sustainable malaria control, and Palestine became, in effect, a ‘laboratory’ for such an attempt.

These ‘ideal’ conditions included, obviously, the presence of holo-endemic malaria, Palestine being described in 1919 by a future director of the London School of Hygiene and Tropical Medicine, Dr. Manson-Bahr, as one of the most highly malarious countries in the world [5]. But Palestine also provided a further ‘ideal’ condition – in 1922, it was as a whole, thinly populated, many rural areas being either almost empty or uninhabitable on account of the disease.

The seriousness of Palestine’s level of malaria was not in doubt. Attempts before WWI by Jewish Zionists to settle in Palestine, their ancient homeland, had been beaten back due to the severity of malaria in the country. In 1913, even the acclaimed international medical journal, the *Lancet*, had published an article confirming the Zionist failure to settle in Palestine on account of the disease [6].

In 1918, over half the British Army in Palestine began to either die or collapse from malaria after exposure to infected mosquitoes for only 10 days whilst fighting the Turkish Army in the final year of WWI.

Such were the conditions in Palestine when Dr. I. Kligler, a brilliant public health scientist, a Jewish Zionist, arrived in 1920 to settle in Palestine, and who was to become the architect of the subsequent successful malaria elimination campaign.

These ‘ideal’ conditions, however, also initially presented a puzzle. There was difficulty at first in understanding how a sparsely-settled country, Palestine, could have such disease because malaria, so widely spread and continuous, was usually correlated with crowding.

It is sometimes overlooked that Palestine was also ‘the Holy Land’, a focus of attention for three different faiths – Judaism, Christianity and Islam – and accordingly for centuries had attracted thousands of pilgrims. After a study by Kligler of the socio-economic conditions prevailing there, he answered the question of why sparsely-settled Palestine suffered with such severe malaria. Bedouins, traditionally nomadic Arab tribes and often living in empty arid regions, travelled through Palestine. Kligler pointed out that the country was small and undeveloped with a constant active movement of the various population groups including the Bedouin but also, significantly, those on annual religious pilgrimages. This movement was as effective in spreading malaria and maintaining its epidemicity as it would be in the case of any other infectious diseases [7].

It is here that the experimental aspect of Kligler’s attempt for sustainable malaria control began. Kliger was to basically apply the same principles employed by the 1918 British Army of destruction of the mosquito breeding sites and ensuring the sites remained unsuitable for propagating anophelines thereafter. In 1917, General Allenby, commander of the British Army in Egypt had previously stressed the necessity for controlling the disease as essential. He already knew of the severity of malaria in Palestine. As commander of an army, he was in a position to order all the necessary steps to be taken to protect his troops from the disease before the final battle he had planned against the Turkish Army. Allenby had previously ordered all anti-malaria work be treated as an absolute priority, and which work included maintenance - ensuring the mosquito sites remained destroyed - whilst the troops prepared for the final advance against the Turkish Army. But subsequently, immediately the advance began, all maintenance of the anti-malaria work ceased as it had served its purpose and thereafter the disease quickly returned.[8] Importantly, the 1918 Allenby experience had also demonstrated that all anti-malaria work had to be carried out thoroughly. Going forward, this was borne in mind at every stage by Kligler, and all procedures and steps he planned were only to be implemented if Kligler had previously felt reassured all stages of the work were likely, not just possibly, to be conducted thoroughly. It was this thoroughness that the ‘laboratory’ would test. Failure to achieve thoroughness in any anti-malaria work meant the work was often pointless, a waste of time.

## The birth of community engagement

The concept ‘community engagement’ may be readily recognised or understood today in 2026, but, in 1922, it was totally unknown. This must be appreciated whilst reading the rest of this paper.

Like Allenby, Kligler was driven by a core focus to succeed in controlling the disease. Control of the disease had to be a priority for Kligler (as it was also for Allenby) otherwise the Zionist project of a Jewish Homeland in Palestine would likely have been impossible. But unlike Allenby’s need to only temporarily control the disease, Kligler had to ensure the disease was sustainably controlled indefinitely.

However, in 1922, Kligler had neither the manpower nor the finances that had been available to Allenby. Allenby not only had his army, if necessary, but he also had over 60,000 labourers, mostly from the Egyptian Labour Corps, to undertake and maintain the anti-malaria work pending the British Army’s attack on 19th September 1918. Probably, as if to warn the Zionists if they intended to attempt to settle in Palestine, a subsequent Army Report stated:

*“It is interesting to speculate on what can be the future of a country such as this from the health point of view. One cannot conceive the problem which faced the Army last spring being undertaken by a Civil Authority. The expense alone would be prohibitive.”*

The Report further added the manpower and the expense referred to treatment of only one strip of country for one year, as if to emphasise the enormity of the problem in attempting similar work for the whole country.

Incidentally, the Army Report had also commented that if no anti-malaria work had been carried out, the troops ‘would have simply melted away’! [9]. Allenby was in a position to have ordered any work to be undertaken, no matter how difficult or disagreeable. Further, after inspection, he could have ordered the task to be repeated if not carried out thoroughly at first.

Kligler, on the other hand, had to ensure that any task was more likely than not carried out willingly, also thoroughly, and all without the necessity of coercion.

## Aspects tested in the ‘laboratory’

### a. Study and investigation preceded any action.

Kligler always arranged for a systematic study of the local aspects of the malaria problem before any work would begin. Such investigation preceded action. The exact direction of the anti-malaria work could only be determined after a thorough as well as comprehensive study of the problem. The characteristic feature was that investigation preceded control, and control preceded any settlement. He decided he was not to be constrained from taking a particular direction merely because previous malaria control had usually taken a different route.

### b. Willing, cooperative manpower.

The only pool of labour Kligler realised he could call upon for manpower was the inhabitants, but there were so very few then. Since the end of the nineteenth century, malaria has been known as a local problem due to the fact that mosquitoes travel only a limited distance from their breeding sites in order to bite and feed on humans. If that mosquito has previously been infected by biting a human already infected, when it bites and feeds on a human, it also injects the malaria parasite at that moment, thereby transmitting the disease.

Kligler reflected upon the problem and decided upon a fresh and untried approach by dealing with the actual control as a local problem. He planned to attend to one locality at a time, and then slowly proceed from that locality to another, destroying mosquito breeding sites in each locality as he went but, importantly, also ensuring that each breeding site was likely to remain destroyed for years to come. Kligler took the unusual step for that time of educating each and every inhabitant in that locality to have an understanding and appreciation of the necessity for the breeding sites to remain destroyed. He maintained that the education of the inhabitants was as important, perhaps even more so, as the destruction of the breeding sites [10].

Kligler was clearly ahead of his time in regard to engagement with the community, and also of an appreciation of its importance in malaria control. Eminent experienced scientists dealing with malaria have recently suggested that 20% of solutions to malaria elimination involve technology, knowledge and science, and that the remaining 80% of solutions involve ‘improved management and better engagement with the community’ [11]. The scientific opinion has therefore recognised that the major part of malaria elimination solution is bound up with community engagement.

There was an obvious advantage for Kligler that Palestine was so thinly populated, and because of such small numbers, he was able to arrange for effective education of all the inhabitants. Such education was sometimes even conducted on a one-to-one basis in cases where an inhabitant had not understood at first how malaria was contracted or what steps were necessary to control the disease. Also, because Kligler tended not to delegate, he initially would often personally attend to inspect or supervise. Therefore, the existence of fewer inhabitants made Kligler’s task that much easier.

A Palestine 1922 Census [12] revealed a rural majority of only 389,534 for the whole country, and even then with such a tiny population, the census also revealed 41 habitually spoken languages. A Palestinian identity or entity did not exist. With such a diversity of cultures and backgrounds, there could have been no blanket approach to such education, often rendering individual education anyway almost impossible to avoid.

### c. Sustainability, reliance on ‘community engagement’

Kligler set out to have all the inhabitants feeling they somehow ‘owned’ the malaria control work in their own locality, that they should feel involved. Limited funds had been collected by the Zionists in the USA, enabling Kligler to offer to contribute part of the cost of the initial work of destruction of mosquito breeding sites on condition that the inhabitants in the locality affected contributed the rest either in money or in kind (e.g., labour). This procedure helped to awaken the interest of the inhabitants in their malaria problem. The maintenance, ensuring the breeding sites remained destroyed, was tedious and arduous over the years but Kligler found he could rely upon the full enthusiastic cooperation in his anti-malaria work of all inhabitants, Jews and Arabs, to defeat the disease. Without this co-operation, Kligler’s attempts to control the disease would have been pointless.

Therefore, instead of viewing the problem from the outset as one gigantic project, he decided to break it up. He would deal with malaria as a local problem, moving slowly from one location to the next, but only after first dealing with the local troublesome mosquito breeding sites and ensuring the local inhabitants understood the necessity for maintenance. Kligler wrote in 1925:

*“The future is plain sailing. As the waste uncultivated areas are resettled, as the waters of the numerous springs now running to waste are harnessed for use, as the people learn to irrigate intelligently, the sources of the Anopheles mosquitoes will increasingly disappear, and malaria will assume its place among the insignificant diseases of Palestine.”* [13].

## Suggestions why these events in 1922 were, and even today are, overlooked, underexploited or forgotten

Kligler died from a heart attack in 1944, but his control method continued and in 1967, malaria was declared eliminated in Israel by WHO.

Kligler had been confronted by major obstacles in 1922, but these were successfully overcome. He modelled his method according to his limited finances, and he was not reliant on drugs, insecticides or bednets.

There was a likelihood Kligler’s ‘community engagement’ would have been frowned upon by some in a world that was then still very colonial. The inhabitants were treated with dignity and respect, evidenced by the attention and time Kligler and his team would have given them. The importance that Kligler attached to education was demonstrated by the fact, if he had felt anyone had not understood, he would return and repeat the exercise. It is true that because there were so few inhabitants, Kligler could have had the time to educate so well, but it did prove the weightiness and very great value of that real, genuine, sincere (not just some token, mechanical) education in malaria control when considering the essential outcome, namely the worth of the inhabitants’ ongoing, continuous involvement in maintenance. Kligler always maintained that education was as important, even perhaps more so, as the anti-malaria work itself.

A further suggestion of why the method has been overlooked is the following possibility. It may also have been the case that 100+ years ago, Zionist attempts and schemes to make Palestine habitable were viewed as ridiculous, absurd or even a little mad. A 1917 Zionist pamphlet [14] described how the world appeared to view Zionist attempts to render Palestine habitable for settlement. To the outside world, the magnitude of the undertaking in Palestine was such that all suggested solutions were impossible. The world viewed Zionist hopes and efforts as a Utopian dream, and out of touch with reality. In 1904, the Zionist Organisation had previously instituted the Olive Tree Fund (subsequently the Tree Fund) in order to raise funds for the work of afforestation for the improvement it causes in the climate of the country. The Tree Fund was the first national movement in the world to attempt afforestation in a semi-arid zone. But climate change, or environmental damage were not then considered at all or were not fashionable as they are now. This was almost sixty years before the existence of today’s main environment organisations (World Wildlife Fund – 1961; Greenpeace - 1971; Green Party - 1972; Friends of the Earth - 1971) and was clearly way ahead of its time.

The Zionists must have appeared a little ‘crazy’ for planting trees in such locations, and perhaps that is how the world may have viewed Kligler’s work.

News of the progress of Kligler’s work, both scientific and practical, was so encouraging that in 1925 the Malaria Commission of the Health Section of the League of Nations [15] visited the country in order to see at close range the work that was going on. The Commission experts were generally impressed with the control, but they described it in their subsequent report as a ‘huge scientific experiment’ and doubted that any government would follow the lead shown by Kligler’s approach.

As if to emphasise its attitude, only two years later, in 1927, the League of Nations issued a report on the Work of the Malaria Commission:

*"... the sole purpose of the campaign against malaria, as far as the Commission is concerned, is to reduce the incidence and severity of the disease. Measures designed to accomplish more than that (particularly measures aiming at eradication) are not a wise proposition ... The Commission had occasion to enter into relations with a number of states which ... had undertaken an energetic campaign against malaria. The disease yielded to the measures which were taken, but reappeared with added virulence as soon as these measures were somewhat relaxed. This is a very serious result which brings in its train disillusion and discouragement.”* [16]

Kligler’s antimalarial method was new and untested. Whilst not mentioning Kligler by name, the report appeared pessimistic about the Palestine inhabitants continuing to maintain the works to ensure the breeding sites remained non-productive or destroyed. The suggestion was that Kligler’s community engagement wouldn’t last, but this pessimistic suggestion with time was proven to be incorrect.

## What today’s malaria control can learn from the 1922 malaria control

By WHO’s own admission, use of insecticides and drugs created a new battleground in the fight against the disease. It is worth returning to the 2012 WHO Handbook mentioned at the start of this paper:

*"Before the Second World War, vector control was conducted predominantly by environmental control of the proliferation of mosquitoes. [e.g., the 1922 Palestine method] .... There is evidence that environmental management had a clear impact on disease; however, elimination of disease was never on the agenda. The advent of DDT and other organochlorine pesticides during the 1940s changed this situation. ... The focus of vector control on insecticides meant that environmental management and other alternative methods were underexploited or even forgotten.”* [4].

Due to insecticide resistance, the fight against the disease has now returned to a similar situation that Kligler could possibly have recognised.

Social and Behaviour Change (SBC) initiatives appear to have become central to today’s attempts for malaria control, shifting focus from merely providing tools (like bednets) to trying to ensure communities actually use them correctly. These evidence-based strategies address the psychological, cultural, and social barriers to prevention, testing, and treatment, and in theory are also intended to encourage the consistent, year-round use of Insecticide-Treated Nets (ITNs) rather than using them only when mosquito nuisance is intense or for other purposes (like fishing or fencing).

Today’s methods are not locally based and instead whilst these attempts are usually based on the use of ITNs and indoor residual-spraying (IRS), the methods in recent years have met with disappointing progress which has slowed and stalled [17]. Today’s methods usually rely upon each of the individual inhabitants using a net correctly and accepting their houses to be sprayed, the weakness being the need to strictly have many (and preferably all) partaking in using nets correctly or having homes sprayed, otherwise control will likely fail. A further weakness is that any inspector is completely dependent on a shaky potentially-unreliable account provided by each inhabitant as to how a net was used the previous night. With today’s methods, there seems to be no way to factually check thoroughness, it is difficult to verify accuracy, so one wonders how progress towards malaria elimination can expect to be planned with such potentially unreliable data.

With Kligler’s method, however, once the Palestine breeding sites had been destroyed, the local inhabitants would have been expected to assume responsibility for maintenance of the anti-malaria work to ensure these sites remained destroyed. The task that was set for each inhabitant had to be reasonably manageable, and Kligler always planned each task for the inhabitants with that requirement in mind to reduce the risk of inhabitants skipping steps. The maintenance had to be carried out correctly and thoroughly, and for which task the inhabitant had been educated. Unlike today’s methods, the Palestine maintenance work was always capable of being visually checked to ensure it had been carried out properly.

Education today is sometimes treated as merely providing an awareness of the disease with the hope that increased awareness can be converted somehow into actual behaviour change. But today’s malaria control methods usually stop there, and leave the job half done. The 1922 method, on the other hand, provided the inhabitants in a locality with a collective path forward, namely to repeatedly ensure that every destroyed breeding site remained destroyed. Due to Kligler’s emphasis on thorough education, inhabitants understood the necessity of repeating the maintenance, the reward of course being eventual malaria elimination.

Finally, there seems to be hardly any emphasis on thoroughness when dealing with control of the disease with current methods. It would appear methods involved in malaria management are not seen today as a priority either by the funder at the top or by the enduser at the bottom. And for this reason, malaria is likely to remain a threat or perhaps even worsen. Absence of resolute determinism to end malaria merely results in evolution catching up with us time and again through drug and insecticide resistance with devastating consequences.

## The irrelevance of only few inhabitants in relation to the lessons of the 1922 malaria-control

It may be incorrectly considered that Kligler’s approach could be relevant only to locations with few inhabitants. Palestine has been referred to in this paper as a laboratory only because Kligler was afforded the opportunity of testing the effectiveness of each step without distraction and without the pressure of great numbers all at the same time clamouring for his attention. Kligler proved malaria could be dealt with as a local problem, and the principle with each step can also be applied elsewhere.

Kligler’s method and approach were successful and as a result, the numbers of inhabitants quickly increased. The Jewish inhabitants increased of course by natural increase and also by immigration. But the number of Arab inhabitants increased dramatically, achieving, at one stage, the highest rate of natural increase in the world.

Whilst there were those mentioned above who were negative and pessimistic about the likelihood of sustainability (or perhaps who didn’t care for Kligler’s anti-colonial approach), there was also applause from eminent experts who had inspected the Palestine work and sensed a successful way forward.

In 1925, Dr. F. Russell, director of the Rockefeller International Health Division inspected the antimalaria work in Palestine, and wrote:

*"I do not know when I have seen better and more successful anti-malaria work than that which is being done in Palestine... The co-operation of the people with the authorities leaves nothing to be desired... It is an ideal way to carry out malaria work because it makes the population served by the antimalaria measures participants in the project from the beginning; they then have a better understanding of the problem and will be more ready than they otherwise would be to take care of the necessary maintenance."* [18].

Also, in 1925, Professor Nocht, President of the Malaria Commission of the League of Nations that came to inspect the works at the end of their two-week tour of inspection stated:

*“Palestine showed the fruits of an energetic and victorious campaign which would stimulate others to follow the methods there employed… It was not the custom of the Commission to make comparisons but he would on this occasion, say that the interest that Palestine had provided was unsurpassed by that of any of their other visits* [to other countries]*, and never had the Commission met with such preparations as had there been provided.”* [19]

Also, one only has to refer to the British Mandates 1941 Malaria Review to appreciate just how successful the 1922 method actually was. If ever proof was needed of a population’s commitment to be free of malaria, then the co-operation that existed in Palestine is that proof. In 1941, the British Mandate’s Department of Health had reviewed the malaria position in Palestine, and praised the cooperation:

*“In the true rural areas the lasting effects of swamp and marsh reclamation, the proper irrigation of gardens and similar areas, and the periodical canalisation, filling and clearing of stream beds, have so impressed the people by the resulting improvement in health, and the land thus made available to farmer and shepherd, that their prompt and energetic cooperation is one of the most remarkable features of the antimalarial campaign in this country.”* [9]

## Conclusion

Kligler’s initial advice to the malaria community today would likely have been to always conduct a systematic study of the local aspects of the malaria problem before any work would begin. The study should be not only of the local terrain, the environment and mosquitoes, but include also the local inhabitants and their backgrounds. It is important to appreciate that local conditions and situations are not necessarily identical with conditions in neighbouring localities. Each locality is potentially a new, fresh malaria problem requiring its own new solutions..

Kligler’s further advice would have probably been to introduce very much greater resources than exist today for real, genuine education, and also to greatly emphasise thoroughness at every step as essential in malaria control.

Hopefully, the principles demonstrated in the 1922 malaria-control ‘laboratory’ will no longer be either underexploited or forgotten!
